# 7,14-Bis(4-bromo­phen­yl)-2,11,11-trimethyl-1,4,10,12-tetra­oxa­dispiro­[4.2.5.2]penta­decane-9,13-dione

**DOI:** 10.1107/S1600536809014251

**Published:** 2009-04-30

**Authors:** Ju-hua Peng, Ning Ma, Ge Zhang

**Affiliations:** a Lianyungang Teachers’ College, Lianyungang 222006, People’s Republic of China; b School of Chemistry and Chemical Engineering, Xuzhou Normal University, Xuzhou 221116, People’s Republic of China

## Abstract

In the mol­ecule of the title compound, C_26_H_26_Br_2_O_6_, the cyclo­hexane ring is in a chair conformation, while the five-membered and 1,3-dioxane rings both adopt envelope conformations. The dihedral angle between the benzene rings is 77.21 (3)°. In the crystal structure, weak inter­molecular C—H⋯O inter­actions link the mol­ecules into centrosymmetric dimers, forming *R*_2_^2^(14) ring motifs. One of the Br atoms, the methyl C and H atoms, and the C atom bonded to the methyl group of the five-membered ring are disordered over two positions. The Br atoms were refined with occupancies of 0.51 (4) and 0.49 (4), while the C and H atoms were refined with occupancies of 0.320 (18) and 0.680 (18).

## Related literature

For general background, see: Davidson & Bernhard (1948 ▶); Meldrum (1908 ▶); Muller *et al.* (2005 ▶); Ramachary *et al.* (2003 ▶); Tietze & Beifuss (1993 ▶); Tietze *et al.* (2001 ▶). For related structures, see: Chande & Khanwelkar (2005 ▶); Ramachary & Barbas (2004 ▶). For bond-length data, see: Allen *et al.* (1987 ▶). For ring-puckering parameters, see: Cremer & Pople (1975 ▶). For graph-set notation, see: Bernstein *et al.* (1995 ▶).
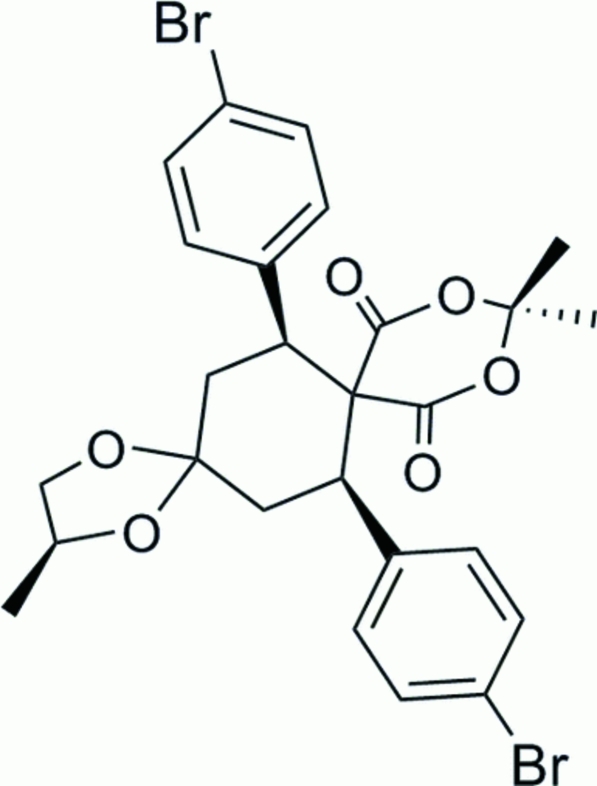


## Experimental

### 

#### Crystal data

C_26_H_26_Br_2_O_6_
*M* *_r_* = 594.29
Triclinic, 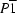

*a* = 7.356 (3) Å
*b* = 12.590 (5) Å
*c* = 14.852 (6) Å
α = 69.787 (6)°
β = 87.415 (7)°
γ = 79.243 (6)°
*V* = 1267.8 (9) Å^3^
*Z* = 2
Mo *K*α radiation
μ = 3.24 mm^−1^
*T* = 298 K
0.18 × 0.11 × 0.09 mm

#### Data collection

Bruker SMART CCD area-detector diffractometer
Absorption correction: multi-scan (*SADABS*; Sheldrick, 1996 ▶) *T*_min_ = 0.594, *T*_max_ = 0.759
6454 measured reflections
4330 independent reflections
1413 reflections with *I* > 2σ(*I*)
*R*_int_ = 0.060

#### Refinement

*R*[*F* ^2^ > 2σ(*F* ^2^)] = 0.073
*wR*(*F* ^2^) = 0.146
*S* = 1.00
4330 reflections
327 parameters
H-atom parameters constrained
Δρ_max_ = 0.44 e Å^−3^
Δρ_min_ = −0.32 e Å^−3^

### 

Data collection: *SMART* (Bruker, 1998 ▶); cell refinement: *SAINT* (Bruker, 1998 ▶); data reduction: *SAINT*; program(s) used to solve structure: *SHELXS97* (Sheldrick, 2008 ▶); program(s) used to refine structure: *SHELXL97* (Sheldrick, 2008 ▶); molecular graphics: *ORTEP-3 for Windows* (Farrugia, 1997 ▶) and *PLATON* (Spek, 2009 ▶); software used to prepare material for publication: *SHELXL97*.

## Supplementary Material

Crystal structure: contains datablocks global, I. DOI: 10.1107/S1600536809014251/hk2666sup1.cif

Structure factors: contains datablocks I. DOI: 10.1107/S1600536809014251/hk2666Isup2.hkl

Additional supplementary materials:  crystallographic information; 3D view; checkCIF report

## Figures and Tables

**Table 1:** Hydrogen-bond geometry (Å, °)

*D*—H⋯*A*	*D*—H	H⋯*A*	*D*⋯*A*	*D*—H⋯*A*
C23—H23⋯O5^i^	0.93	2.60	3.286 (2)	131

Symmetry code: (i) 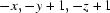.

**Fig. 1.**
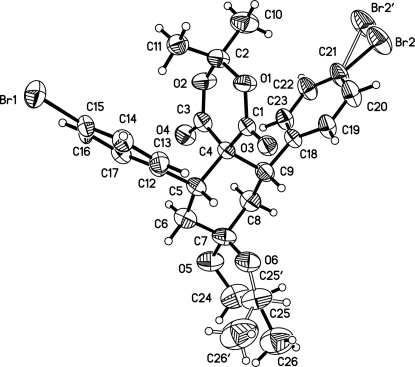
The molecular structure of the title molecule, with the atom-numbering scheme. Displacement ellipsoids are drawn at the 30% probability level.

**Fig. 2.**
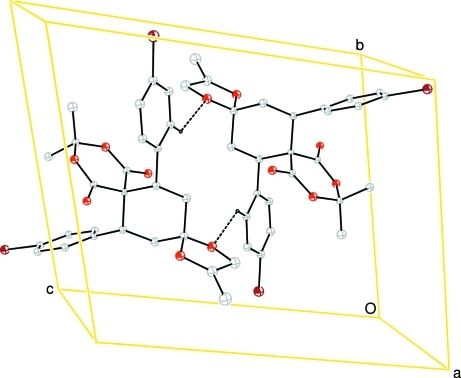
A partial packing diagram of the title compound. Hydrogen bonds are shown as dashed lines.

## supplementary crystallographic information

### Comment

Over the past few decades, Meldrum's acid (2,2-dimethyl-1,3-dioxane-4,6-dione) (Tietze & Beifuss, 1993; Tietze *et al.*,  2001) has been used as a versatile organic reagent (Ramachary *et al.*,  2003; Muller *et al.*, 2005) and its derivatives are very useful building blocks in synthetic organic chemistry (Davidson & Bernhard, 1948; Meldrum, 1908). Spirocyclic compounds including a Meldrum's acid unit are attractive intermediates in the syntheses of natural products and in medicinal chemistry. Thus, the synthesis of new highly substituted spiro ring system with a Meldrum's acid unit has attracted widespread attention (Ramachary & Barbas, 2004; Chande & Khanwelkar, 2005). We report herein the crystal structure of the title compound.

In the molecule of the title compound (Fig. 1), the bond lengths (Allen *et al.*,  1987) and angles are within normal ranges. Ring A (C4-C9) is not planar, having total puckering amplitude, Q_T_, of 0.576 (3) Å and chair conformation [φ = -150.30 (3) and θ = 3.77 (3) °] (Cremer & Pople, 1975). Rings D (C12-C17) and E (C18-C23) are, of course, planar, and they are oriented at a dihedral angle of D/E = 77.21 (3)°. Rings B (O1/O2/C1-C4) and C (O5/O6/C7/C24/C25) adopt envelope conformations, with atoms C2 and O5 displaced by -0.184 (3) and -0.291 (3) Å from the planes of the other rings atoms.

In the crystal structure, weak intermolecular C-H···O interactions (Table 1) link the molecules into centrosymmetric dimers forming R_2_^2^(14) ring motifs (Fig. 2) (Bernstein *et al.*,  1995), in which they may be effective in the stabilization of the structure.

### Experimental

The title compound was prepared by the reaction of 1,2-diarylidenehydrazine (2 mmol), Meldrum's acid (5 mmol), HOAc (4 mL) and (S)-1,2-propanediol (8 ml). Upon completion, the reaction mixture was cooled to room temperature, and introduced into water. The solid was collected by washing with water. The combined solid was purified by ethanol.

### Refinement

The Br2, C25, H25, C26, H26A, H26B and H26C atoms were disordered. During the refinement process, the disordered C and H atoms were refined with occupancies of 0.320 (18) and 0.680 (18), while Br atoms were refined with occupancies of 0.51 (4) and 0.49 (4). H atoms were positioned geometrically, with C-H = 0.93, 0.98, 0.97 and 0.96 Å for aromatic, methine, methylene and methyl H, respectively, and constrained to ride on their parent atoms, with U_iso_(H) = xU_eq_(C), where x = 1.5 for methyl H and x = 1.2 for all other H atoms.

### Crystal data

**Table:**

C_26_H_26_Br_2_O_6_	*Z* = 2
*M**_r_* = 594.29	*F*(000) = 600
Triclinic, *P*1	*D*_x_ = 1.557 Mg m^−^^3^
Hall symbol: -P 1	Mo *K*α radiation, λ = 0.71073 Å
*a* = 7.356 (3) Å	Cell parameters from 920 reflections
*b* = 12.590 (5) Å	θ = 2.6–25.3°
*c* = 14.852 (6) Å	µ = 3.24 mm^−^^1^
α = 69.787 (6)°	*T* = 298 K
β = 87.415 (7)°	Prism, colorless
γ = 79.243 (6)°	0.18 × 0.11 × 0.09 mm
*V* = 1267.8 (9) Å^3^	

### Data collection

**Table:**

Bruker SMART CCD area-detector diffractometer	4330 independent reflections
Radiation source: fine-focus sealed tube	1413 reflections with *I* > 2σ(*I*)
graphite	*R*_int_ = 0.060
φ and ω scans	θ_max_ = 25.0°, θ_min_ = 1.9°
Absorption correction: multi-scan (*SADABS*; Sheldrick, 1996)	*h* = −8→4
*T*_min_ = 0.594, *T*_max_ = 0.759	*k* = −14→14
6454 measured reflections	*l* = −17→16

### Refinement

**Table:**

Refinement on *F*^2^	Secondary atom site location: difference Fourier map
Least-squares matrix: full	Hydrogen site location: inferred from neighbouring sites
*R*[*F*^2^ > 2σ(*F*^2^)] = 0.073	H-atom parameters constrained
*wR*(*F*^2^) = 0.146	*w* = 1/[σ^2^(*F*_o_^2^) + (0.038*P*)^2^] where *P* = (*F*_o_^2^ + 2*F*_c_^2^)/3
*S* = 1.00	(Δ/σ)_max_ < 0.001
4330 reflections	Δρ_max_ = 0.44 e Å^−^^3^
327 parameters	Δρ_min_ = −0.32 e Å^−^^3^
Primary atom site location: structure-invariant direct methods	

### Special details

**Table:**

Geometry. All e.s.d.'s (except the e.s.d. in the dihedral angle between two l.s. planes) are estimated using the full covariance matrix. The cell e.s.d.'s are taken into account individually in the estimation of e.s.d.'s in distances, angles and torsion angles; correlations between e.s.d.'s in cell parameters are only used when they are defined by crystal symmetry. An approximate (isotropic) treatment of cell e.s.d.'s is used for estimating e.s.d.'s involving l.s. planes.
Refinement. Atom C25 is disordered over two sites, C25 and C25', for which occupation factors were refined and converged to 0.320 (18) and 0.680 (18), respectively. Atom Br2 is disordered over two sites, Br2 and Br2', for which occupation factors were refined and converged to 0.51 (4) and 0.49 (4), respectively

### Fractional atomic coordinates and isotropic or equivalent isotropic displacement parameters (Å^2^)

**Table:**

	*x*	*y*	*z*	*U*_iso_*/*U*_eq_	Occ. (<1)
Br1	−0.42012 (15)	0.06501 (8)	1.10942 (7)	0.0968 (5)	
Br2	0.153 (4)	0.9617 (10)	0.6351 (8)	0.129 (4)	0.51 (4)
Br2'	0.052 (4)	0.9718 (7)	0.6478 (10)	0.133 (4)	0.49 (4)
O1	0.0177 (8)	0.4886 (5)	0.8942 (4)	0.0710 (15)	
O2	−0.2101 (7)	0.5229 (5)	0.7764 (4)	0.0800 (16)	
O3	0.2839 (8)	0.3774 (4)	0.9013 (4)	0.0636 (16)	
O4	−0.1776 (7)	0.4473 (4)	0.6644 (4)	0.0771 (17)	
O5	0.2593 (9)	0.2562 (5)	0.5545 (4)	0.1010 (19)	
O6	0.4502 (10)	0.2393 (5)	0.6762 (4)	0.0924 (17)	
C1	0.1367 (14)	0.4189 (7)	0.8619 (6)	0.057 (2)	
C2	−0.1683 (13)	0.5300 (8)	0.8662 (7)	0.067 (2)	
C3	−0.1111 (14)	0.4561 (7)	0.7342 (7)	0.068 (2)	
C4	0.0841 (11)	0.4000 (6)	0.7706 (6)	0.0578 (18)	
C5	0.1176 (11)	0.2655 (6)	0.7948 (5)	0.0652 (19)	
H5	0.2411	0.2357	0.8250	0.078*	
C6	0.1253 (13)	0.2337 (7)	0.7058 (6)	0.084 (2)	
H6A	0.0047	0.2600	0.6740	0.100*	
H6B	0.1540	0.1505	0.7239	0.100*	
C7	0.2707 (14)	0.2862 (7)	0.6355 (7)	0.082 (2)	
C8	0.2277 (12)	0.4198 (7)	0.6086 (6)	0.081 (2)	
H8A	0.3187	0.4532	0.5636	0.098*	
H8B	0.1068	0.4498	0.5772	0.098*	
C9	0.2306 (11)	0.4533 (6)	0.6926 (6)	0.069 (2)	
H9	0.3536	0.4200	0.7227	0.083*	
C10	−0.2069 (13)	0.6528 (7)	0.8562 (6)	0.106 (3)	
H10A	−0.3337	0.6847	0.8365	0.158*	
H10B	−0.1274	0.6932	0.8089	0.158*	
H10C	−0.1845	0.6603	0.9167	0.158*	
C11	−0.2822 (11)	0.4550 (6)	0.9417 (5)	0.076 (2)	
H11A	−0.4112	0.4804	0.9241	0.114*	
H11B	−0.2618	0.4611	1.0029	0.114*	
H11C	−0.2454	0.3763	0.9454	0.114*	
C12	−0.0149 (12)	0.2112 (6)	0.8688 (6)	0.0561 (19)	
C13	0.0441 (12)	0.1672 (6)	0.9644 (6)	0.068 (2)	
H13	0.1648	0.1672	0.9807	0.081*	
C14	−0.0782 (13)	0.1233 (6)	1.0353 (6)	0.072 (2)	
H14	−0.0391	0.0948	1.0993	0.087*	
C15	−0.2547 (13)	0.1210 (6)	1.0128 (7)	0.064 (2)	
C16	−0.3171 (11)	0.1642 (6)	0.9171 (6)	0.067 (2)	
H16	−0.4377	0.1640	0.9009	0.080*	
C17	−0.1928 (13)	0.2069 (6)	0.8477 (6)	0.069 (2)	
H17	−0.2311	0.2340	0.7836	0.083*	
C18	0.2047 (12)	0.5829 (6)	0.6711 (6)	0.061 (2)	
C19	0.3090 (12)	0.6205 (7)	0.7226 (6)	0.078 (2)	
H19	0.4012	0.5686	0.7645	0.094*	
C20	0.2792 (13)	0.7354 (7)	0.7134 (6)	0.093 (3)	
H20	0.3513	0.7617	0.7481	0.111*	
C21	0.1409 (15)	0.8104 (7)	0.6517 (7)	0.085 (3)	
C22	0.0427 (13)	0.7738 (7)	0.5975 (6)	0.081 (2)	
H22	−0.0453	0.8257	0.5530	0.097*	
C23	0.0739 (12)	0.6600 (7)	0.6087 (6)	0.072 (2)	
H23	0.0037	0.6344	0.5725	0.086*	
C24	0.4398 (14)	0.2316 (9)	0.5185 (7)	0.130 (3)	
H24A	0.4661	0.2965	0.4645	0.156*	
H24B	0.4542	0.1642	0.4994	0.156*	
C25	0.5598 (15)	0.2100 (9)	0.6036 (8)	0.119 (3)	0.320 (18)
H25	0.5957	0.2858	0.5774	0.142*	0.320 (18)
C26	0.755 (3)	0.147 (3)	0.602 (2)	0.160 (9)	0.320 (18)
H26A	0.8223	0.1402	0.6583	0.240*	0.320 (18)
H26B	0.8157	0.1889	0.5459	0.240*	0.320 (18)
H26C	0.7525	0.0714	0.6009	0.240*	0.320 (18)
C25'	0.5598 (15)	0.2100 (9)	0.6036 (8)	0.119 (3)	0.680 (18)
H25'	0.6610	0.2537	0.5858	0.142*	0.680 (18)
C26'	0.633 (3)	0.0856 (14)	0.6492 (12)	0.201 (8)	0.680 (18)
H26D	0.6963	0.0725	0.7082	0.302*	0.680 (18)
H26E	0.7168	0.0596	0.6068	0.302*	0.680 (18)
H26F	0.5318	0.0441	0.6624	0.302*	0.680 (18)

### Atomic displacement parameters (Å^2^)

**Table:**

	*U*^11^	*U*^22^	*U*^33^	*U*^12^	*U*^13^	*U*^23^
Br1	0.0912 (9)	0.0934 (8)	0.1038 (8)	−0.0385 (6)	0.0252 (6)	−0.0232 (6)
Br2	0.173 (10)	0.061 (2)	0.150 (3)	−0.016 (4)	−0.045 (5)	−0.030 (2)
Br2'	0.173 (10)	0.047 (2)	0.170 (4)	−0.003 (4)	−0.049 (6)	−0.027 (2)
O1	0.065 (4)	0.081 (3)	0.077 (3)	0.006 (3)	−0.006 (3)	−0.048 (3)
O2	0.070 (4)	0.094 (3)	0.079 (3)	0.013 (3)	−0.007 (3)	−0.047 (3)
O3	0.058 (4)	0.055 (3)	0.076 (4)	−0.003 (3)	−0.012 (3)	−0.022 (3)
O4	0.070 (4)	0.078 (4)	0.084 (4)	−0.002 (3)	−0.025 (3)	−0.032 (3)
O5	0.110 (4)	0.106 (4)	0.085 (4)	0.006 (3)	0.006 (3)	−0.043 (3)
O6	0.106 (4)	0.095 (4)	0.086 (4)	−0.005 (3)	0.007 (3)	−0.049 (3)
C1	0.059 (4)	0.055 (4)	0.066 (4)	−0.015 (4)	0.003 (4)	−0.028 (3)
C2	0.059 (5)	0.078 (5)	0.072 (5)	0.008 (5)	−0.022 (5)	−0.042 (4)
C3	0.075 (4)	0.060 (4)	0.074 (4)	−0.017 (4)	−0.005 (4)	−0.027 (4)
C4	0.059 (4)	0.054 (3)	0.069 (4)	−0.012 (3)	0.003 (3)	−0.031 (3)
C5	0.073 (4)	0.062 (4)	0.072 (4)	−0.014 (3)	0.004 (4)	−0.037 (3)
C6	0.095 (4)	0.070 (4)	0.087 (4)	−0.008 (4)	0.002 (4)	−0.032 (4)
C7	0.094 (5)	0.081 (4)	0.078 (5)	0.001 (4)	0.005 (4)	−0.046 (4)
C8	0.090 (4)	0.077 (4)	0.078 (4)	−0.016 (4)	0.001 (4)	−0.028 (4)
C9	0.080 (4)	0.060 (4)	0.074 (4)	−0.018 (3)	0.006 (4)	−0.028 (3)
C10	0.107 (7)	0.088 (6)	0.116 (7)	0.019 (6)	−0.015 (6)	−0.044 (6)
C11	0.067 (6)	0.090 (6)	0.082 (6)	−0.012 (5)	−0.002 (5)	−0.045 (5)
C12	0.055 (4)	0.054 (4)	0.072 (4)	−0.016 (4)	0.001 (4)	−0.035 (3)
C13	0.063 (5)	0.061 (4)	0.080 (5)	−0.015 (4)	0.002 (4)	−0.024 (4)
C14	0.074 (6)	0.062 (4)	0.082 (5)	−0.016 (4)	0.003 (5)	−0.024 (4)
C15	0.063 (5)	0.056 (4)	0.079 (5)	−0.024 (4)	0.004 (5)	−0.026 (4)
C16	0.057 (5)	0.066 (5)	0.089 (6)	−0.034 (4)	0.003 (5)	−0.028 (4)
C17	0.081 (5)	0.061 (4)	0.071 (4)	−0.016 (4)	−0.007 (4)	−0.027 (4)
C18	0.072 (4)	0.053 (4)	0.064 (4)	−0.016 (4)	−0.003 (4)	−0.026 (3)
C19	0.085 (5)	0.064 (4)	0.089 (5)	−0.027 (4)	−0.012 (4)	−0.021 (4)
C20	0.108 (6)	0.064 (5)	0.104 (5)	−0.025 (5)	−0.037 (5)	−0.017 (5)
C21	0.106 (6)	0.045 (4)	0.101 (6)	−0.021 (5)	−0.022 (5)	−0.013 (4)
C22	0.098 (6)	0.049 (4)	0.088 (5)	−0.010 (4)	−0.030 (5)	−0.011 (4)
C23	0.083 (5)	0.057 (4)	0.075 (4)	−0.021 (4)	−0.009 (4)	−0.016 (4)
C24	0.113 (6)	0.148 (6)	0.118 (6)	0.004 (6)	0.001 (6)	−0.044 (5)
C25	0.106 (5)	0.123 (5)	0.106 (5)	0.021 (5)	0.010 (4)	−0.037 (4)
C26	0.156 (15)	0.138 (14)	0.150 (14)	0.013 (14)	0.011 (14)	−0.026 (13)
C25'	0.106 (5)	0.123 (5)	0.106 (5)	0.021 (5)	0.010 (4)	−0.037 (4)
C26'	0.179 (13)	0.184 (12)	0.180 (12)	0.009 (11)	0.044 (11)	−0.011 (11)

### Geometric parameters (Å, °)

**Table:**

Br1—C15	1.870 (8)	C11—H11A	0.9600
Br2—C21	1.853 (12)	C11—H11B	0.9600
Br2'—C21	1.997 (15)	C11—H11C	0.9600
O1—C1	1.316 (9)	C12—C17	1.374 (10)
O1—C2	1.397 (9)	C12—C13	1.391 (10)
O2—C3	1.313 (9)	C13—C14	1.386 (9)
O2—C2	1.419 (8)	C13—H13	0.9300
O3—C1	1.193 (9)	C14—C15	1.363 (10)
O4—C3	1.213 (9)	C14—H14	0.9300
O5—C7	1.390 (8)	C15—C16	1.400 (10)
O5—C24	1.428 (9)	C16—C17	1.380 (9)
O6—C7	1.411 (9)	C16—H16	0.9300
O6—C25	1.426 (9)	C17—H17	0.9300
C1—C4	1.534 (10)	C18—C23	1.355 (10)
C2—C10	1.475 (10)	C18—C19	1.356 (10)
C2—C11	1.525 (10)	C19—C20	1.381 (10)
C3—C4	1.510 (11)	C19—H19	0.9300
C4—C5	1.577 (9)	C20—C21	1.376 (11)
C4—C9	1.604 (9)	C20—H20	0.9300
C5—C12	1.503 (9)	C21—C22	1.347 (10)
C5—C6	1.504 (9)	C22—C23	1.360 (10)
C5—H5	0.9800	C22—H22	0.9300
C6—C7	1.535 (10)	C23—H23	0.9300
C6—H6A	0.9700	C24—C25	1.492 (10)
C6—H6B	0.9700	C24—H24A	0.9700
C7—C8	1.562 (10)	C24—H24B	0.9700
C8—C9	1.450 (9)	C25—C26	1.512 (16)
C8—H8A	0.9700	C25—H25	0.9800
C8—H8B	0.9700	C26—H26A	0.9600
C9—C18	1.526 (9)	C26—H26B	0.9600
C9—H9	0.9800	C26—H26C	0.9600
C10—H10A	0.9600	C26'—H26D	0.9600
C10—H10B	0.9600	C26'—H26E	0.9600
C10—H10C	0.9600	C26'—H26F	0.9600
			
C1—O1—C2	126.5 (8)	H11A—C11—H11C	109.5
C3—O2—C2	126.0 (7)	H11B—C11—H11C	109.5
C7—O5—C24	110.3 (8)	C17—C12—C13	118.4 (8)
C7—O6—C25	105.9 (8)	C17—C12—C5	123.7 (8)
O3—C1—O1	119.5 (9)	C13—C12—C5	117.8 (8)
O3—C1—C4	122.3 (9)	C14—C13—C12	119.5 (8)
O1—C1—C4	118.1 (8)	C14—C13—H13	120.3
O1—C2—O2	113.3 (7)	C12—C13—H13	120.3
O1—C2—C10	106.9 (8)	C15—C14—C13	121.1 (9)
O2—C2—C10	107.4 (7)	C15—C14—H14	119.4
O1—C2—C11	107.3 (7)	C13—C14—H14	119.4
O2—C2—C11	107.5 (7)	C14—C15—C16	120.5 (8)
C10—C2—C11	114.8 (8)	C14—C15—Br1	120.7 (8)
O4—C3—O2	118.2 (10)	C16—C15—Br1	118.8 (7)
O4—C3—C4	122.9 (9)	C17—C16—C15	117.4 (8)
O2—C3—C4	118.8 (9)	C17—C16—H16	121.3
C3—C4—C1	114.0 (8)	C15—C16—H16	121.3
C3—C4—C5	111.8 (7)	C12—C17—C16	123.0 (9)
C1—C4—C5	106.2 (6)	C12—C17—H17	118.5
C3—C4—C9	110.4 (7)	C16—C17—H17	118.5
C1—C4—C9	104.9 (6)	C23—C18—C19	119.1 (8)
C5—C4—C9	109.2 (6)	C23—C18—C9	123.1 (8)
C12—C5—C6	114.8 (6)	C19—C18—C9	117.7 (8)
C12—C5—C4	111.2 (6)	C18—C19—C20	120.4 (9)
C6—C5—C4	112.0 (6)	C18—C19—H19	119.8
C12—C5—H5	106.0	C20—C19—H19	119.8
C6—C5—H5	106.0	C21—C20—C19	118.7 (9)
C4—C5—H5	106.0	C21—C20—H20	120.6
C5—C6—C7	112.4 (7)	C19—C20—H20	120.6
C5—C6—H6A	109.1	C22—C21—C20	120.7 (8)
C7—C6—H6A	109.1	C22—C21—Br2	126.7 (9)
C5—C6—H6B	109.1	C20—C21—Br2	111.0 (10)
C7—C6—H6B	109.1	C22—C21—Br2'	115.6 (9)
H6A—C6—H6B	107.9	C20—C21—Br2'	123.3 (9)
O5—C7—O6	108.0 (7)	Br2—C21—Br2'	22.3 (3)
O5—C7—C6	107.4 (8)	C21—C22—C23	119.1 (9)
O6—C7—C6	110.3 (8)	C21—C22—H22	120.4
O5—C7—C8	110.5 (7)	C23—C22—H22	120.4
O6—C7—C8	111.3 (8)	C18—C23—C22	121.8 (9)
C6—C7—C8	109.1 (7)	C18—C23—H23	119.1
C9—C8—C7	111.6 (7)	C22—C23—H23	119.1
C9—C8—H8A	109.3	O5—C24—C25	101.7 (8)
C7—C8—H8A	109.3	O5—C24—H24A	111.4
C9—C8—H8B	109.3	C25—C24—H24A	111.4
C7—C8—H8B	109.3	O5—C24—H24B	111.4
H8A—C8—H8B	108.0	C25—C24—H24B	111.4
C8—C9—C18	114.6 (7)	H24A—C24—H24B	109.3
C8—C9—C4	112.0 (6)	O6—C25—C24	109.5 (9)
C18—C9—C4	109.8 (6)	O6—C25—C26	133.7 (15)
C8—C9—H9	106.6	C24—C25—C26	116.0 (13)
C18—C9—H9	106.6	O6—C25—H25	93.0
C4—C9—H9	106.6	C24—C25—H25	93.0
C2—C10—H10A	109.5	C26—C25—H25	93.0
C2—C10—H10B	109.5	C25—C26—H26A	109.5
H10A—C10—H10B	109.5	C25—C26—H26B	109.5
C2—C10—H10C	109.5	H26A—C26—H26B	109.5
H10A—C10—H10C	109.5	C25—C26—H26C	109.5
H10B—C10—H10C	109.5	H26A—C26—H26C	109.5
C2—C11—H11A	109.5	H26B—C26—H26C	109.5
C2—C11—H11B	109.5	H26D—C26'—H26E	109.5
H11A—C11—H11B	109.5	H26D—C26'—H26F	109.5
C2—C11—H11C	109.5	H26E—C26'—H26F	109.5

### Hydrogen-bond geometry (Å, °)

**Table:**

*D*—H···*A*	*D*—H	H···*A*	*D*···*A*	*D*—H···*A*
C23—H23···O5^i^	0.93	2.60	3.286 (2)	131

Symmetry codes: (i) −*x*, −*y*+1, −*z*+1.
